# Lifespan trajectories of risk preference, impulsivity, and self-control: A dataset containing self-report, informant-report, behavioral, hormone and functional neuroimaging measures from a cross-sectional human sample

**DOI:** 10.1016/j.dib.2023.109968

**Published:** 2023-12-15

**Authors:** Loreen Tisdall, Simon Mugume, David Kellen, Rui Mata

**Affiliations:** a Center for Cognitive and Decision Sciences, Faculty of Psychology, University of Basel, Switzerland; b College of Arts & Sciences, Syracuse University, USA

**Keywords:** Aging, Risk taking, Propensity, Task, Balloon analogue risk task, Delay discounting, Mixed gambles, Testosterone, fMRI

## Abstract

This paper describes data collected from a cross-sectional convenience sample of 200 healthy human volunteers between 16 and 81 years of age. We assembled an extensive battery of measures of risk preference, impulsivity, and self-control, as well as a range of demographic and cognitive measures, Crucially, we adopted different measure categories, including self-reports, informant reports, behavioral measures, and biological measures (hormones, brain function) to capture individual differences, and adopted a within-participant design. Data collection took place over multiple sessions. First, participants completed a laboratory session at the university during which we collected computer-assisted self-report measures (i.e., standardized questionnaires) as well as behavioral measures using computerized tasks. Second, participants independently completed a home session that included the completion of self-report measures, and the collection of saliva samples. In parallel, we acquired informant reports from up to three individuals nominated by the study participants. Third, participants completed a final session at the local hospital during which we collected structural and functional neuroimaging data, as well as further self-report measures. The data was collected to address questions concerning the developmental trajectories of risk preference and related constructs while assessing the impact of the assessment method; however, we invite fellow researchers to benefit from and further explore the data for research on decision-making under risk and uncertainty in general, and to apply novel analytical approaches (e.g., machine-learning applications to the neuroimaging data). Combining a large set of measures with a within-participant design affords a wealth of opportunities for further insights and a more robust evidence base supporting current theorizing on (age-related) differences in risk preference, impulsivity, and self-control.

Specifications Table

**Table:** 

Subject	Experimental and Cognitive Psychology, Neuropsychology and Physiological Psychology
Specific subject area	Developmental trajectories of risk preference, impulsivity, and self-control
Type of data	Comma-separated values files (.csv) Portable Document Format (.pdf) JavaScript Object Notation files (.json) Tab-separated values files (.tsv) Compressed Neuroimaging Informatics Technology Initiative files (nifti.gz)
How the data were acquired	Self-report measures, well-being, and demographic variables were collected using the online survey platform UniPark, as well as pen-and-paper questionnaires. Informant reports were collected using pen-and-paper questionnaires. We used E-Prime software to collect behavioral measures (including a cognitive battery). Participants independently collected six saliva samples. The neuroimaging data were collected on a 3T Siemens MAGNETOM Prisma MRI system with the 20-channel head coil. During scanning, we collected an anatomical scan and functional scans for two behavioral measures. Behavioral data from the functional neuroimaging sequences were collected using E-Prime.
Data format	Raw data Partly analyzed Transformed Filtered
Description of data collection	We collected data from a cross-sectional convenience sample of 200 healthy human adults between 16 and 81 years of age. Participants completed a laboratory session, a home session, and a magnetic resonance imaging (MRI) session. We also collected informant reports from up to three individuals nominated by study participants.
Data source location	Institution: University of Basel City/Town/Region: Basel, Basel-Stadt Country: Switzerland
Data accessibility	**Self-report, informant-report, behavioral, and hormone measures** Repository name: Open Science Framework Direct URL to data: https://osf.io/sh34n/
	**Neuroimaging data** Repository name: OpenNeuro Data identification number: ds004711 Direct URL to data: https://openneuro.org/datasets/ds004711
Related research article	This paper is not a companion paper to empirical work published elsewhere. However, we have analyzed subsets of the data, and have reported these analyses in a published paper as well as a manuscript currently available as a preprint: Tisdall, L., & Mata, R. (2023). Age differences in the neural basis of decision-making under uncertainty. Cognitive, Affective, & Behavioral Neuroscience, 1–21. 10.3758/s13415-022-01060-6 Tisdall, L., Frey, R., Wulff, D.U., Kellen, D., & Mata, R. (preprint). Developmental Trajectories of Risk Preference, Impulsivity, and Self-Control: A Multiverse Approach. 10.31219/osf.io/uj359

## 1 Value of the Data

• The dataset results from an effort to understand lifespan trajectories of risk preference and consists of a large age-heterogenous sample of individuals, covering several modalities (self-report, informant-report, behavioral tasks, hormone, neuroimaging) on standardized measures of risk preference, impulsivity, and self-control, as well as a cognitive function battery (working-memory, semantic memory, numeracy). The dataset is unique in combining the breadth of measurement modalities for these constructs in an age-heterogenous sample.
• The current data may be of interest to researchers from various disciplines, including (but not limited to) cognitive scientists, psychologists, neuroscientists, and economists interested in individual and age differences in decision-making under risk and uncertainty, reward processing, temporal discounting, impulsivity, self-control, personality, and cognitive function.
• This dataset supports robust science by inviting independent efforts to examine the primary study goals (i.e., convergence and divergence of developmental trajectories as a function of the construct and assessment method), potentially adopting alternative or more sophisticated techniques than those planned at data collection.
• Possible (re)use of the data includes individual and age differences analyses of cognitive function (working memory, semantic memory, numeracy), model-based analysis of informant reports, comparative computational modeling of behavioral tasks, and advanced processing of and extracting indices from functional neuroimaging data (e.g., multivariate pattern analysis).
• Further (re)use cases involve comprehensive research synthesis efforts, for example using individual participant data meta-analysis concerning any of the measures included in the study.

## 2 Objective

Conceptual clarification and the development of theories are central challenges for psychological science. In the field of psychological science of aging, extant theories and methods aim to describe and explain the link between aging and individual differences in decision-making under risk and uncertainty. However, existing work is characterized by the unsystematic examination of pertinent constructs (e.g., risk preference, impulsivity, and self-control), as well as the uncoordinated adoption of assessment methods (e.g., self-report, informant-report, behavioral, or biological measures). This state of affairs has contributed to a heterogeneous evidence base concerning the lifespan trajectories of risk preference and related constructs. The overarching aim of this dataset was to facilitate the extensive examination of trajectories of risk preference and related constructs across the adult human lifespan and to probe the convergence of trajectories across different measure categories rooted in distinct measurement traditions.

## 3 Data Description

In what follows we provide more detailed descriptions of the actual data. To achieve maximum transparency and legibility given the extensive number of data points collected for every participant, we organize this section by the repository in which the data is deposited. All measures are further documented and described in Section 4 (Experimental design, materials, and methods) of this paper.

### 3.1 Open Science Framework (OSF)

**Fig. 1:**
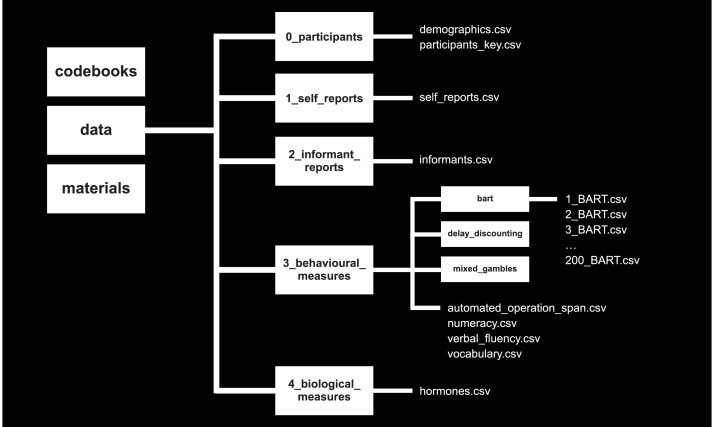
Schematic overview of the structure of the Open Science Framework repository.

As outlined in Fig. 1, our OSF repository is organized into three parent directories: ‘codebooks’, ‘data’, and ‘materials’. To facilitate the linkage of files across directories (e.g., codebooks with relevant data files), we applied consistent naming conventions. For example, in each of the three parent directories we created child directories that are consistently labeled 0_participants’, ‘1_self_reports’, ‘2_informant_reports’, ‘3_behavioral_measures’, ‘4_biological_measures’ (note that we only deposit data and materials associated with the hormone analyses on OSF; the neuroimaging data are deposited on OpenNeuro). All codebooks are provided as PDF documents and contain detailed descriptions of all variables contained in the data files. All data files are provided as CSV files, their contents, and variables being meticulously documented in the codebooks.

All data files contain a 5-character participant ID variable to facilitate the merging of different data files by participant ID. In the data subdirectory labeled ‘0_participants’ we provide demographic data (i.e., ‘demographics.csv’) for each participant, and in the respective codebook (i.e., ‘Codebook_demographics.pdf) we detail the respective scales and response options. Critically, we provide a data file ‘participants_key.csv’ which can be used to match, for example, the self-report and behavioral data on OSF with the neuroimaging data on OpenNeuro. Where possible, we provide one file that contains data for all participants (e.g., demographics, self-reports, informant reports, hormones, as well as for four behavioral measures, namely numeracy, verbal fluency, vocabulary, and automated operation span). However, the extent of three of the behavioral measures (namely Balloon Analogue Risk Task, delay discounting, and mixed gambles) required data to be provided as individual data files, that is, one data file per participant. For each of these three tasks, we provide one folder containing individual CSV files and also provide extensive codebooks to facilitate future analyses. All data were collected in Switzerland; thus, in the directory ‘materials’, we make available the original wordings (in German) of each item used for self-reports and informant reports, as well as the mathematical problems used in the numeracy task. The data file for the vocabulary task (‘vocabulary.csv’) contains both the individual (German) words presented as stimuli as well as participants’ responses.

### 3.2 OpenNeuro

**Fig. 2:**
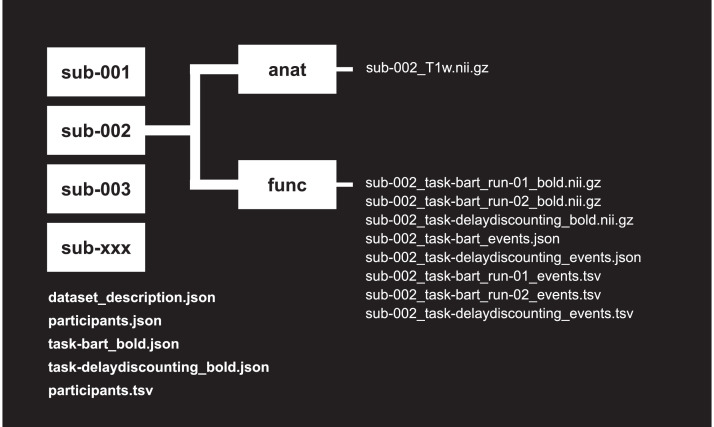
Schematic overview of the structure of the neuroimaging dataset on OpenNeuro.

As shown in Fig. 2, the neuroimaging data deposited on OpenNeuro was organized and labeled according to the Brain Imaging Data Structure (https://bids.neuroimaging.io). In total we scanned 187 participants, all of whom completed the Balloon Analogue Risk Task (BART). Out of the 187 participants, 178 also completed the delay discounting task inside the scanner (due to time constraints we were unable to collect delay discounting data for nine participants). We provide accompanying ‘.json’ and ‘.tsv’ files to detail and describe the participants, tasks, and pertinent acquisition parameters. For example, the file ‘participants.tsv’ contains participants’ id, gender, and age, as well as three columns (‘bart_run1’, ‘bart_run2’, ‘delay_discounting’) indicating whether a participant has functional neuroimaging data for two runs of the BART and the delay discounting task, respectively. Importantly, while the ID contained in this file is specific to the neuroimaging session, this id can be linked up with self-report, informant-report, behavioral, and hormone data using the key (‘participants_key.csv’) provided on OSF. The variables contained in ‘participants.tsv’ are described in detail in the accompanying ‘participants.json’ file.

The individual-level neuroimaging data are organized into 189 folders, with subfolders for anatomical (‘anat’) and functional (‘func’) data. (Note that we included empty subdirectories for the two participants for which we did not collect MRI data, as this was thought to facilitate automated preprocessing pipelines). For every participant with MRI data, the ‘anat’ folder contains a T1 structural image (e.g., ‘sub_002_T1w.nii.gz’) that was processed (see Section 4.6.4 for further details and functions) to safeguard the anonymity of participants by stripping non-brain tissue (e.g., bone, eyes, teeth). Each participant's ‘func’ folder contains task-specific functional brain images; two images for the BART (run 1 and run 2), and if collected, one image for delay discounting. Similar to the structural images, the functional images were processed to remove any non-brain tissue from the individual images. In addition, for every functional brain image, we provide the associated events and their onsets (e.g., ‘sub-002_task-bart_run-01_events.tsv’), and also provide codebooks (e.g., ‘sub-002_task-bart_events.json’) for the BART and delay discounting event files in every participant's ‘func’ folder.

## 4 Experimental Design, Materials and Methods

In the next sections we provide detailed descriptions of participant recruitment, the data collection protocol, as well as all study materials and methods. Where required, we provide relevant references for materials and methods.

### 4.1 Recruitment, screening, and final sample

We recruited a cross-sectional convenience sample from the local community using advertisements, public academic presentations, and the lab participant database. Advertisements included a project-specific email address and telephone number to allow individuals to express their interest in study participation. Trained study personnel then contacted interested individuals to introduce the study in more detail, and to conduct an initial screening to determine individuals’ eligibility for study participation. Screening covered initial questions such as individuals’ age and overall well-being, but also covered essential inclusion and exclusion criteria for participation in the neuroimaging session. This comprehensive screening process was conducted to identify any factors that may pose risks in relation to magnetic resonance imaging (MRI) safety. Individuals who reported having permanent implants (e.g., pacemakers, cochlear implants, neurostimulators, insulin pumps), as well as those who experienced claustrophobia, tinnitus, epilepsy, or had permanent metal objects inside or on their bodies (such as surgical clips, metal splinters, metal prostheses, copper coils, or artificial heart valves), or had previously undergone heart or brain surgery, were excluded from participation. Additionally, individuals who reported conditions that would prevent them from lying comfortably still inside the scanner, those who were pregnant, or those who reported the use of prescribed medications that could interfere with cognitive and neural function were also excluded.

Resulting from this recruitment process was a final sample of 200 healthy human volunteers (51% female) between the ages of 16 and 81 years (Mean age = 45.0, SD = 19.0), with a comparable age distribution for the 102 female participants (Mean age = 44.5, SD = 19.1) and 98 male participants (Mean age = 45.6, SD = 20.0) included in the study. A detailed description of the final sample is provided in Table 1. All participants provided written informed consent prior to participation in this study.

**Table 1:** Overview of sample demographics.

	*N* = 200
Mean age in years (SD)	45.0 (19.0)
Gender female (%)	102 (51)
Marital status (%)	
*Single*	68 (34.0)
*In relationship*	45 (22.5)
*Married*	67 (33.5)
*Divorced*	14 (7.0)
*Widowed/partner deceased*	5 (2.5)
*Registered partnership*	0 (0)
*Resolved registered partnership*	1 (0.5)
Education (%)	
*No degree/certificate*	0 (0.0)
*Primary school*	1 (0.5)
*Secondary/district/orientation school*	13 (6.5)
*10th grade, 1-year pre-apprenticeship, 1-year vocational school*	7 (3.5)
*Diploma secondary school, traffic school, technical secondary school*	6 (3.0)
*Vocational apprenticeship, vocational school, vocational baccalaureate*	42 (21.0)
*Secondary school, grammar school baccalaureate, seminary*	34 (17.0)
*Higher technical/vocational training, higher technical college*	27 (13.5)
*University of applied sciences, HWV, technical school*	12 (6.0)
*University, college, ETH/Poly*	58 (29.0)
Children (%)	
*None*	111 (55.5)
*One*	27 (13.5)
*Two*	46 (23.0)
*Three*	11 (5.5)
*Four*	4 (2.0)
*Five or more*	1 (0.5)
Main occupation (%)	
*In education*	55 (27.5)
*Employed*	81 (40.5)
*Unpaid work / activity*	6 (3.0)
*Unemployed*	7 (3.5)
*Retired*	51 (25.5)
Native language (%)*	
*(Swiss) German*	180 (90.0)
*French*	8 (4.0)
*Italian*	4 (2.0)
*Spanish*	4 (2.0)
*English*	16 (8.0)
*Other*	20 (10.0)
Personal monthly gross income in Swiss Francs (%)	
*<=1000*	39 (19.5)
*1001–3000*	46 (23.0)
*3001–5000*	37 (18.5)
*5001–7000*	38 (19.0)
*7001–10,000*	16 (8.0)
*10,001–15,000*	11 (5.5)
*>=15,001*	5 (2.5)
*NA*	8 (4.0)
Total value of assets in Swiss Francs (%)	
*<=1000*	20 (10.0)
*1001–5000*	22 (11.0)
*5001–10,000*	20 (10.0)
*10,001–20,000*	18 (9.0)
*20,001–30,000*	14 (7.0)
*30,001–50,000*	16 (8.0)
*50,001–100,000*	24 (12.0)
*>=100,001*	60 (30.0)
*NA*	6 (3.0)
Total value of debt in Swiss Francs (%)	
*<=1000*	110 (55.0)
*1001–5000*	10 (5.0)
*5001–10,000*	7 (3.5)
*10,001–20,000*	4 (2.0)
*20,001–30,000*	2 (1.0)
*30,001–50,000*	3 (1.5)
*50,001–100,000*	3 (1.5)
*>=100,001*	26 (13.0)
*NA*	35 (17.5)

⁎ *Note:* Some participants indicated several native languages, thus frequencies and percentages do not add up to 200 and 100%, respectively. NA = missing, no response provided by participants.

### 4.2 Data collection protocol

Data collection took place at the Center for Cognitive and Decision Sciences at the University of Basel, as well as the University Hospital. The collection of data from the study participants was split across three separate sessions (Table 2). First, participants completed a laboratory session, during which we collected computer-assisted self-report measures (i.e., standardized questionnaires) as well as behavioral measures using computerized tasks. Second, participants independently completed a home session that included the completion of self-report measures, and the collection of saliva samples. Third, participants completed a final session at the local hospital during which we collected neuroimaging data and the final set of self-report measures. For the majority of participants, all three sessions took place over the course of two to three weeks. All study participants were provided with financial reimbursement of 15 Swiss Francs (∼15 US Dollars) per hour of participation, resulting in a maximum participation fee of 98 Swiss Francs for 6.5 h of participation. Payments were made after each completed session, which allowed for timely and partial payments in case of early termination of participation. Performance in the behavioral measures included in both the laboratory and the neuroimaging session was also incentivized, thus adding to the overall monetary payment received.

**Table 2:** Overview of the data collection sessions.

	Screening	Laboratory session	Home session	MRI session	Informants
Duration	20–30 min	180 min	90 min	120 min	30 min
Informed consent		✓		✓	✓
Demographic data	✓	✓		✓	✓
Self-report measures		✓	✓	✓	
Behavioral measures		✓		✓	
Biological measures			✓hormones	✓ brain	
Informant-report measures		✓ nomination			✓
Cognitive battery		✓			

To gather informant reports, participants were asked during the laboratory session to nominate up to three individuals whom they believed could serve as informants. Once nominated, each person was sent a study packet by mail which contained written study information, a consent form, a printed survey, a labeled return envelope, and a voucher valued at 10 Swiss Francs (approximately 11 US Dollars) as compensation for their time; this could be redeemed at local shops and amenities.

### 4.3 Neuroimaging data acquisition

Neuroimaging data were collected at the University Hospital Basel using a Siemens 3T MAGNETOM Prisma MRI system equipped with a 20-channel head coil. At the beginning of the MRI session, we obtained a structural T1-weighted scan for each participant using a magnetization-prepared rapid gradient echo sequence. The parameters for this scan were as follows: repetition time (TR) of 2500 ms, echo time (TE) of 4.25 ms, inversion time (TI) of 1100 ms, flip angle of 7°, field of view of 256 mm × 256 mm, 192 slices, and voxel dimensions of 1.0 mm isotropic. For the task-related functional runs, we acquired T2*-weighted blood-oxygen-level-dependent (BOLD) echo-planar images for each person. The acquisition parameters for these images were as follows: repetition time (TR) of 2010 ms, echo time (TE) of 30 ms, flip angle of 78°, field of view of 192 mm × 192 mm, voxel size of 3 mm × 3 mm × 3 mm, and 33 transversal slices per volume with a 15% distance factor. The MRI acquisition protocol can be accessed via the OSF repository.

### 4.4 Materials

To capture individual differences in risk preference and related constructs, we assembled an extensive battery of measures and adopted different measure categories, including self-reports, informant reports, behavioral measures, and biological measures (hormones, brain function). The data was collected in Switzerland; thus, all materials and instructions were presented in German. Whenever possible, we provide the materials used in this study on OSF, as well as English translations. For the translations, we used the web-based DeepL Translator (https://www.deepl.com/translator) to translate each item from German to English. The translations were checked by a native English speaker and, if necessary, adjusted for comprehension. For ease of access and use, the self-report and informant-report items that require recoding have already been recoded (see OSF documentation).

#### 4.4.1 Self-report measures

To address the main objective of the project for which the dataset was collected, we adopted an extensive set of self-report measures (Table 3) to capture individual differences in risk preference and risky activities, as well as related constructs (e.g., impulsivity, self-control). Self-reports were also used to capture other constructs, including personality and affect, and to assess general health, well-being and demographic variables. Some of the questionnaires were adopted twice to allow for test-retest analyses (albeit over a short time interval of approximately two to three weeks) or to allow for state versus trait assessment of affect. For context, we report published test-retest reliabilities for the self-report measures (Table 3), and cite the source of the estimate. To note, test-retest reliability varies as a function of the exact implementation (e.g., test-retest interval), sample (size) and scoring procedure (e.g., total score versus subscale score) [1]; to document and differentiate between these is beyond the scope of this paper.

**Table 3:** Overview of self- and informant-report measures.

Scale	Included in self-reports	Assessment session self-reports	Included in informant reports	Test-retest reliability [Ref.]	Ref.
General risk-taking propensity	✓	Laboratory, MRI	✓	0.7 [11]	[2,3]
Domain-specific risk-taking propensity (recreation, trust)	✓	Laboratory, MRI	✓	0.58–0.62 [11]	[2,3]
Domain-Specific Risk-Taking (DOSPERT) Scale	✓	Home		0.42–0.81 [1]	[11,12]
Frequency of risky behaviors (4 and 12 months)	✓	Home		0.51–0.88 [11]	[11]
Behavioral Inhibition / Behavioral Activation Scale	✓	MRI	✓	0.44–0.82 [1]	[7,13]
Barratt Impulsiveness Scale	✓	Laboratory	✓	0.23–0.92 [1]	[4,14]
Sensation Seeking Scale	✓	Home	✓	0.52–0.94 [1]	[6,15]
UPPS Impulsive Behavior Scale	✓	Home	✓	0.64–0.93 [1]	[5,16]
Brief Self-Control Scale (German: SCS-K-D)	✓	Laboratory	✓	0.75–0.87 [1]	[8,17]
GRIT Scale (German: BISS)	✓	MRI	✓	0.61–0.78 [1]	[10,18]
Low Self-Control Scale	✓	Home	✓	0.44–0.76 [9]	[9,19]
GSOEP Big Five Inventory	✓	Laboratory		0.57–0.80 [20]	[20,21]
Positive and Negative Affect Schedule (trait and state)	✓	Laboratory & MRI (state), Home (trait)		0.84 [22]	[22,23]
Health Survey (SF-36)	✓	Home		0.60–0.81 [24]	[24,25]
Demographic information (e.g., age, gender, education)	✓	Laboratory	✓	0.95–0.99 [1]	[3]

#### 4.4.2 Informant reports

The informant surveys collected information regarding the informant's age, gender, duration and type of relationship with the study participant, as well as ratings on the study participant’s risk preference, impulsivity, and low self-control. To minimize the burden on informants, we asked them to rate a subset of 54 items that were originally completed by the study participants (Table 3). Specifically, informants provided ratings on the study participant's general risk-taking behavior and domain-specific risk-taking in areas such as recreation/sport and trust in strangers [2,3]. They also rated 12 items (two per factor) from the Barratt Impulsiveness Scale (Version 11) [4], eight items (two per factor) from the UPPS Impulsive Behavior Scale [5], eight items (two per factor, easy and difficult) from the Sensation Seeking Scale [6], four items (two per factor) from the BIS/BAS Scale [7], three items (including the item with the highest loading and two items tied for the second highest loading) from the Brief Self-Control Scale [8], 12 items (two per factor) from the Low Self-Control Scale [9], and four items (two per factor) from the GRIT Scale [10].

#### 4.4.3 Behavioral measures

Apart from the verbal fluency task, all behavioral measures were programmed in E-Prime 2.0 and displayed on a computer screen. Responses were recorded on the keyboard. For two measures (BART, delay discounting) we collected data both outside and inside the MRI scanner; for details about the MRI versions of the task, see Section 4.4.5 ‘Neuroimaging paradigms’.

##### 4.4.3.1 Balloon Analogue Risk Task (BART)

The BART is a commonly used behavioral measure to capture risk taking [26]. In this study, participants aimed to accumulate monetary winnings by inflating virtual balloons without causing them to explode. Successful pumps resulted in earnings, while explosions led to the loss of accumulated earnings for that balloon. Participants were not provided with information about the balloon’s capacity or explosion points; instead, they had to learn and develop a mental understanding of these factors through experience over time. Participants were only informed that the balloon could explode at any point between the first pump and filling up the computer screen. For each successful pump, participants earned 0.05 Swiss Francs, and these earnings accumulated across successful pumps (e.g., four pumps would earn 0.20 Swiss Francs). If participants chose to stop pumping before an explosion occurred, their accumulated earnings for that balloon (e.g., 0.20 Swiss Francs after four pumps) were saved in a permanent account. However, if participants continued pumping and caused an explosion, all accumulated earnings for that balloon were lost, and no additional earnings were saved. Participants completed a total of 40 balloons: 20 with a maximum capacity of 64 pumps and 20 with a maximum capacity of 128 pumps. Participants were informed about two balloon types represented by red and blue colors (colors were randomized between the two capacity conditions for each participant), but the specific capacities were not disclosed. The explosion point for each balloon was determined randomly within a uniform distribution between one and the maximum capacity of that balloon type (i.e., 64 or 128). Feedback on accumulated earnings for the current trial (balloon) and total earnings after the trial was provided on the screen between trials. The sequence and manipulation of explosion points were not controlled between participants. Participants received their accumulated earnings in cash at the end of the laboratory session.

##### 4.4.3.2 Delay discounting

Participants were presented with a delay discounting task using established procedures [27]. Specifically, participants completed two training trials and 80 test trials, all involving decisions between a ‘smaller-sooner’ or ‘larger-later’ amount. The order of the 80 trials was randomized for each participant. These trials encompassed five distinct temporal pairings of ‘smaller-sooner’ versus ‘larger-later’ amounts: today versus in two weeks, today versus in four weeks, in two weeks versus in four weeks, two weeks versus six weeks, and four weeks versus six weeks. Each of these pairings included 16 trials, which varied based on eight possible percentage differences (1, 3, 5, 10, 15, 25, 35, 50) between the smaller and larger amounts. We generated the 80 different magnitudes by randomly selecting numbers from a normal distribution, with the smaller amount ranging from five to 40. To ensure incentive compatibility, participants were informed that one trial would be chosen randomly, and their decision would be realized. If a delayed option was selected, participants received the corresponding amount after the specified waiting time. Payments scheduled for a later time were delivered in cash through registered mail.

##### 4.4.3.3 Mixed gambles

Participants engaged in a series of forced choices between two lotteries characterized by different numbers, magnitudes, and probabilities of monetary outcomes [28]. A total of 210 trials were completed, divided into two runs of 105 trials with a short break in between. The specific lotteries presented during each trial were carefully designed by one of the authors (D.K.) to optimize the computation of loss aversion indices. Each of the seven unique types of gambles was represented by 30 trials, featuring specific combinations of outcome numbers, magnitudes, and probabilities. One type involved participants deciding between two gambles, both with a 50% chance of a gain or an equivalent loss (e.g., Option A: 58 Swiss Francs gain with 50% probability and −58 Swiss Francs loss with 50% probability; Option B: 72 Swiss Francs gain with 50% probability and −72 Swiss Francs loss with 50% probability). In another type, both gambles consisted of three equally likely outcomes (i.e., 33.3%) or outcomes with varying probabilities (e.g., 80%, 10%, and 10%). Importantly, the probabilities of outcomes were identical for the two gambles, with the only distinction being the magnitude of the two or three possible outcomes. Additionally, when two outcome gambles were presented, the gains and losses within each gamble were symmetric (i.e., the same magnitude). For three outcome gambles, two outcomes represented symmetric gains and losses, while the third option remained consistent across the two gambles.

##### 4.4.3.4 Vocabulary

Participants encountered several 5-word sequences [29]. One of the five words was an actual German word, whereas the remaining four words were non-words (in German). Participants were instructed to indicate the actual German word. In total, participants completed 37 different word sequences, and there was no time limit.

##### 4.4.3.5 Numeracy

Participants were given eight scenarios, where each scenario included a different numerical problem [30]. Solving these problems required participants to compute probabilities or frequencies for particular events described in each scenario. There was no time limit for this task.

##### 4.4.3.6 Working memory

Participants completed an automated operation span task which involved 75 numerical exercises to assess working memory [31]. During the encoding stage, on each trial, participants are presented with a math operation (e.g. (1 × 2) + 1 = ?). Participants indicate per mouse click when they have solved the operation, and then continue to the next screen. On the next screen, a digit is presented, which participants must judge to be either the correct or incorrect answer to the math operation encountered on the previous screen. Participants’ judgment of the number as being the correct or incorrect solution is followed by the presentation of a letter for 800 ms. This is followed by a recall stage. During recall, after every four trials of solving math operations, participants are presented with a set of 12 letters, and their task is then to indicate which letters were presented during the previous four trials, as well as the order in which these letters were presented. After recall, feedback is presented for 2000 ms, indicating how many of the four letters were correctly recalled, and how many of the math operations were solved correctly. Participants completed 75 problems, ranging in complexity from three to seven operations and associated letters per set.

##### 4.4.3.7 Verbal fluency

In this free-listing paradigm [32]**,** participants were provided with a category (*animals*) and were then instructed to name (by saying out loud) as many examples of this category as possible (e.g., dog, cat, horse) in a pre-specified time of three minutes. We recorded participants’ spoken (oral) responses as audio files, and provide transcriptions of these audio files (see Section 4.6.2 for details).

#### 4.4.4 Hormone samples

Participants were asked to provide six saliva samples over the course of two consecutive days. Each day, they were instructed to collect three samples: one sample upon awakening, another sample 30–45 min later, and a final sample in the evening. To ensure high-quality samples, participants received both an in-person demonstration and detailed written instructions after the laboratory session. The demonstration and instructions covered the proper method and timing for collecting saliva samples, as well as the correct storage procedures. Additionally, participants had to document their saliva collection on a paper form, which included recording the day, time, and duration since their last food or drink intake for each sample. Participants received salivettes for saliva collection (SaliCap set, IBL Hamburg, Germany), as these were thought to facilitate self-administration. To ensure consistency, all sample containers were pre-labeled with the participant's identification number, collection day (day 1 or day 2), and sample number (1, 2, or 3). Participants were instructed to store the samples in their refrigerator, with all samples being frozen upon receipt by the study personnel. Hormone samples were assayed externally (see Section 4.6.3 for details).

#### 4.4.5 Neuroimaging paradigms

During the MRI session, participants completed two behavioral measures adapted for the scanner: the BART [33] and a delay discounting task [27]. The two fMRI paradigms were comparable to the laboratory versions of the two measures with regards to trial structure, participant instructions, incentivized performance, and the visual presentation, but were optimized for the scanner with regards to stimulus timing and color (see Sections 4.4.5.1 ‘BART fMRI’ and 4.4.5.2 ‘Delay discounting fMRI’ for further details). The stimuli used for the laboratory and the fMRI version of both measures are available on the Open Science Framework (cf. ‘materials’ folder). Before entering the scanner, all participants completed brief training programs for the fMRI versions of the two tasks. The purpose of these training programs was to familiarize participants visually and motorically with the two tasks they would complete during the scanning session. The behavioral measures used inside the scanner were programmed and implemented using E-Prime 2.0, and were synchronized with the scanner trigger signal to ensure the temporal alignment between volume collection and the initiation of behavioral measures. The stimuli were projected onto a screen positioned behind the scanner, and participants could see these through a mirror placed on top of the head coil. To record participants' responses, we used a hand-held Celeritas response system, which involved capturing participants’ button presses made with the right middle and ring finger. As our participant group encompassed individuals with varying ages, MRI-safe glasses were available to compensate for any vision impairments.

##### 4.4.5.1 BART fMRI

For fMRI scanning, we used an adapted version of the BART that included three types of balloons: two reward balloon types (red and blue, counterbalanced across participants) and a gray motor control balloon. The gray balloons served solely as a motor control condition for the fMRI contrast analyses and did not contribute to participants' earnings. In contrast, reward balloons had explosion points randomly selected from a uniform distribution ranging from one to 16. However, they differed in terms of the payoff function used to accumulate rewards for successful pumps. One reward balloon type followed a standard reward function that increased monotonically, meaning that each successful pump resulted in the same gain throughout the entire BART. The other reward balloon type, in contrast, followed an exponential function: the payoff for a successful pump was low (i.e., lower than in the standard reward balloon) at the beginning of the trial, but increased steadily with additional pumps. Each participant completed two runs of the BART inside the scanner, with each run lasting approximately 10 min. Participants were instructed to press a button with their right middle finger to pump a balloon. To stop pumping and collect the earnings from a balloon, they were instructed to press a button with their right ring finger. Feedback regarding earnings for the current trial and the total earnings across all trials, including the current one, was presented on the screen at the end of each trial. A fixation cross visually separated the trials. The fMRI BART employed a controlled temporal sequence with predetermined but randomly selected inter-stimulus intervals between stimulus offset and stimulus onset within a trial (ranging from 1000 to 2000 ms, with a mean of 1500 ms) and inter-trial intervals between trial offset and trial onset (ranging from 1000 to 11,000 ms, with a mean of 4340 ms). Participants controlled the pace of their performance, and the incentives were aligned with their earnings, which were paid out in cash at the end of the fMRI session.

##### 4.4.5.2 Delay discounting fMRI

During the scanner session, participants completed a total of 80 trials, involving five different delay-pairings: ‘today versus in two weeks’, ‘today versus in four weeks’, ‘in two weeks versus in four weeks’, ‘in two weeks versus in six weeks’, and 'in four weeks versus in six weeks'. Additionally, we included eight percentage differences (1, 3, 5, 10, 15, 25, 35, 50) between the amounts of smaller and larger rewards. To determine the reward amounts, we generated 80 random numbers from a normal distribution. The minimum value was set at five, and the maximum was 40, representing the smaller amounts. Within each delay pairing, we added the corresponding percentage difference to the smaller amount to calculate the larger amount. This approach ensured that the specific magnitudes of the smaller and larger rewards varied to avoid repetition from the lab session. During each trial, participants were initially presented with both the smaller-sooner and larger-later offers simultaneously on the screen. They had a maximum of five seconds to make their choice by pressing a button. Specifically, the right middle finger corresponded to choosing the smaller-sooner option, while the right ring finger corresponded to choosing the larger-later option. If no response was recorded within the time limit, the trial ended without a recorded response. After making their choice, participants received visual feedback confirming their chosen option, and the trial concluded. Participants were given an incentive-compatible setup, and their payment was based on one of their randomly chosen decisions. For optimal visibility, we presented the fMRI version of the task with an inverted color format, displaying white information against a black background. Trials were visually separated by a fixation cross. Additionally, the task was programmed to include a mean inter-trial interval of 4320 ms, with intervals ranging from 1000 ms to 11,000 ms.

### 4.5 Missing data

**Fig. 3:**
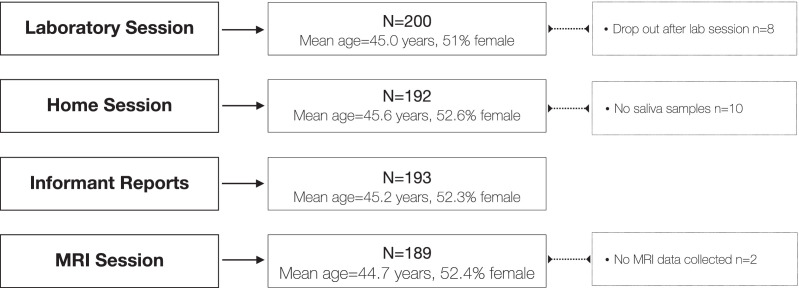
Overview of drop-outs and missing data, organized by data collection session.

As shown in Fig. 3, all 200 recruited participants (100%) completed the laboratory session. Out of these, 192 participants (96%) completed the home session. At the time of the neuroimaging session, 189 participants (94.5%) were still enrolled in the study, and we collected neuroimaging data from 187 participants; two individuals started the session but were unable to enter the scanner due to discomfort. Note that participants are not fully nested in subsequent sessions; for example, participants may have participated in the neuroimaging session but did not complete the home session questionnaires. Regarding collecting informant reports, we received a total of 471 informant reports for 193 out of 200 participants (96.5%). Among these reports, 31 participants (16.1%) had one informant report, 46 participants (23.8%) had two informant reports, and 116 participants (60.1%) had three informant reports, resulting in an average of 2.4 informant reports per participant. Regarding the collection of hormone data, 190 participants (96.5%) provided 1134 saliva samples. From one (0.5%) of those 190 participants we received four samples, from four participants (2.1%) we received five samples, and from 185 participants (97.4%) we received all six saliva samples, averaging 5.7 saliva samples per participant.

### 4.6 Preprocessing and anonymization procedures

Where possible we make the raw data files available. However, for one of the behavioral measures we provide summary statistics due to technical reasons. Moreover, to uphold the principles of open science and safeguard the anonymity of participants, we implemented various anonymization techniques for some of the data discussed in this paper. In the subsequent sections, we outline these procedures.

#### 4.6.1 Computation of scores for the automated operation span task

The data for the automated operan span (Ospan) task were collected using a pre-programmed E-Prime script [31]. To simplify and standardize the analyses given the complexity and sequential nature of the task, the script we used to run the task was set up to provide five summary statistics as part of the output data files for every participant. We provide these five summary statistics for each participant. The summary statistics were obtained according to the following published instructions [27, p. 501]: ‘After the task, the program reported five scores to the experimenter: Ospan score, total number correct, math errors, speed errors, and accuracy errors. The first, the Ospan score, used our traditional absolute scoring method. This was the sum of all perfectly recalled sets. So, for example, if an individual correctly recalled 3 letters in a set size of 3, 4 letters in a set size of 4, and 3 letters in a set size of 5, his or her Ospan score would be 7 (3 4 0). The second score, “total number correct,” was the total number of letters recalled in the correct position. Three types of errors were reported: “Math errors” were the total number of task errors, which were then broken down into “speed errors,” in which the participant ran out of time in attempting to solve a given math operation, and “accuracy errors,” in which the participant solved the math operation incorrectly.’

#### 4.6.2 Transcription of verbal fluency audio files

To protect individuals’ identities, we transcribed all audio files recorded for the verbal fluency task. The data file for this task includes all (task-relevant) utterances produced over the course of the three minutes of the task. We also transcribed repetitions and category-irrelevant words (e.g., ‘safari’ or ‘fur’ for the category ‘animals’), and also transcribed each utterance as faithfully to the original production as possible (e.g., we transcribed singular and plural). We only coded productions as missing in case a word was either incomprehensible or inaudible.

#### 4.6.3 Assaying of saliva sample

After data collection was complete, participants’ saliva samples were sent to an independent external lab (http://www.dresden-labservice.de) for analysis. Aligned with our focus on testosterone, the chosen lab had the specialized expertise and appropriate equipment for analyzing testosterone levels in saliva samples. To maintain integrity during transportation, the samples were shipped on dry ice. At the external lab, the saliva samples underwent a quality control process. Subsequently, we received 1134 testosterone scores (unit = pg/ml) for 190 participants, with each participant having between four and six measurements taken. The external lab also provided a detailed record of all the samples received, including information about the quality of each sample. Additionally, certain samples were double-checked to ensure the accuracy and validity of individuals’ testosterone data. Once the necessary assays were completed, the lab disposed of all the samples, ensuring their proper destruction.

#### 4.6.4 De-identification of neuroimaging data

All structural and functional magnetic resonance neuroimaging data were preprocessed using the FSL Brain Extraction Tool (BET) to remove the skull and any other (potentially identifying) non-brain tissues. Structural images were processed using the function bet, specified as follows: *bet 〈input〉 〈output〉 -f 0.5 -o*. Quality assurance checks suggested that the default threshold of 0.5 was suboptimal for one participant; accordingly, we made adjustments to the threshold for this image and documented the threshold on OpenNeuro. For the functional images, we specified the extraction function as follows: *bet 〈input〉 〈output〉 -F*; this produced good results for all images.

## Ethics Statements

The project for which the data was collected was reviewed and approved by the Ethikkommission Nordwest- und Zentralschweiz EKNZ (EKNZ BASEC 2015-00094). All regulations and ethical guidelines were followed during the conduct of the research. All participants received verbal and written study information and provided written informed consent prior to participation.

## CRediT Author Statement

**Loreen Tisdall:** Conceptualization, Methodology, Software, Investigation, Data Curation, Writing- Original draft preparation, Writing- Reviewing and Editing, Visualization, Supervision, Project administration, Funding acquisition. **Simon Mugume**: Data Curation, Writing- Reviewing and Editing. **David Kellen**: Methodology, Writing- Reviewing and Editing. **Rui Mata**: Conceptualization, Methodology, Writing- Reviewing and Editing, Supervision, Funding acquisition. All authors approved the final version of the submitted manuscript.

## Data Availability

Lifespan trajectories of risk preference, impulsivity, and self-control (Original data) (Open Science Framework). AgeRisk (Original data) (OpenNeuro).
